# Spontaneous Eruption of Premolar Associated with a Dentigerous Cyst

**DOI:** 10.1155/2016/5323978

**Published:** 2016-05-30

**Authors:** Irla Karlinne Ferreira de Carvalho, Anibal Henrique Barbosa Luna

**Affiliations:** Paraiba Federal University (UFPB), 58091-900 João Pessoa, PB, Brazil

## Abstract

Dentigerous cyst (DC) is the second most common odontogenic cyst with greater incidence in young patients. It presents as a unilocular, asymptomatic radiolucency involving the crown of an impacted tooth, commonly noticed in X-rays to investigate absence, wrong tooth position, or delay in the chronology of eruption. Decompression/marsupialization (D/M) is the most implemented treatment, especially when preserving the tooth involved is advised. The aim of this study is to discuss the DC characteristics that contribute to spontaneous eruption of premolars, by reporting the case of a conservative treatment of DC. This eruption depends on factors such as age, angulation of inclusion, rate of root formation, depth of inclusion, and eruption space. This paper reports the case of a 10-year-old patient with a radiolucent lesion diagnosed as DC involving element 35, which erupted as a result of treatment. The patient was observed during 1 year and 6 months.

## 1. Introduction

Dentigerous cysts (DC), also denominated follicular cysts, are one of the two most common types of odontogenic cysts associated with impacted, included, or partially erupted teeth [1].

It is ranked the second most common odontogenic cyst, and the literature has shown an occurrence of approximately 24% among all the true cysts of the maxillae [2]. The majority of reports have shown a peak incidence of this cyst in the second and third decade of life, although some have reported the fifth decade [3].

A typical DC clinically presents as a unilocular, asymptomatic, radiolucent lesion involving the crown of an included or impacted tooth. Generally there is no pain or discomfort associated with it, unless it becomes secondarily infected [2]. The radiolucence generally arises at the cementoenamel junction of the tooth [3].

The large majority are discovered accidentally when radiographs are taken to investigate a failure of eruption or a poorly positioned tooth [2]. In the majority of cases, the diagnosis of a DC is simple. However, differential diagnosis must be made with alterations such as a dental follicle or a hyperplastic dental follicle and even an odontogenic keratocyst or unicystic ameloblastoma [3].

It is formed due to an alteration in the reduced epithelium of the enamel and develops from an accumulation of fluid between the reduced epithelium of the enamel and the crown of the developing tooth [2]. The teeth most frequently affected are the third molars, maxillary canines, and premolars [1].

Enucleation and decompression/marsupialization (D/M) are the forms of treatment most used for DC [4]; the only difference between them is by the use of a device in decompression to maintain communication of the lesion with the oral cavity. Some important criteria must be considered for the treatment plan, such as the size of the cyst, age, proximity to anatomic structures [5], clinical importance, and possibility of making use of the tooth involved.

In spite of the clinical peculiarities of each case and of the method of treatment chosen, these lesions have a favorable prognosis. Therefore, the aim is to report a case of conservative treatment of a dentigerous cyst in a pediatric patient.

## 2. Case Report

**Figure 1:**
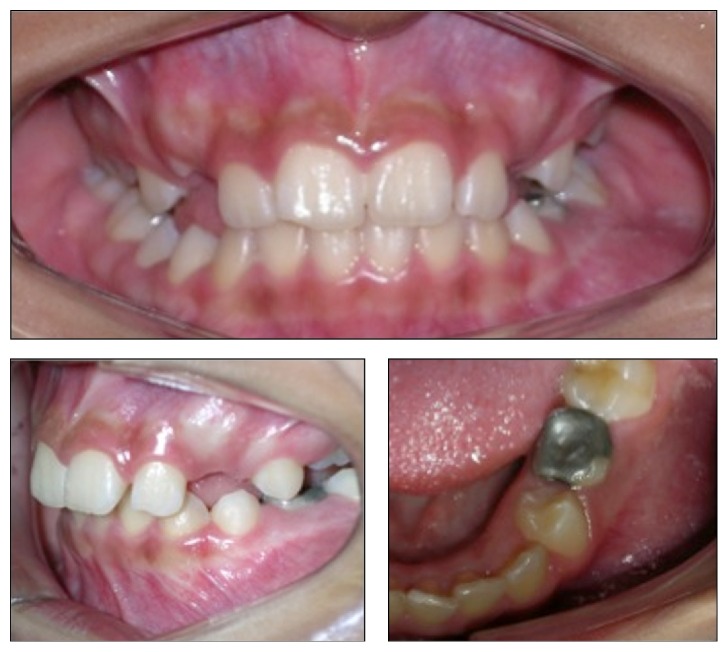
Swelling in the vestibular cortex of the left premolar area and retention of second primary molar, with large amalgam restoration.

**Figure 2:**
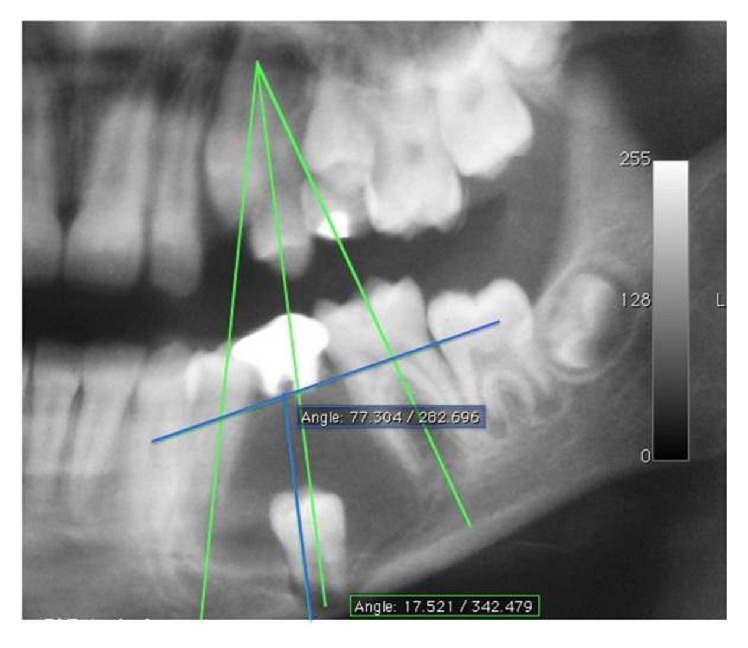
Angulation proposed in the studies of Fujii et al. [11] and Hyomoto et al. [10], to the value of 77.3°, in green. Angulation proposed by Yahara et al. [12], to the value of 17.5°, in blue.

The patient, L. J. S., a 10-year-old boy, mulatto, presented to the Oral and Maxillofacial Clinic of the Lauro Wanderley University Hospital (HULW/UFPB), accompanied by his guardian, complaining of “pain when chewing on the left side.” He presented no facial asymmetry on physical exam; however, on intraoral inspection, left second premolar was observed to be absent, with retention of left second primary molar with a large amalgam restoration showing a mesiodistal dimension of 10 mm. In addition, an increase in volume, firm on palpation and asymptomatic, was found, with a normal aspect of the oral mucosa in the region of the left mandibular body, leading to obliteration of the sulcus (Figure 1). The panoramic radiograph showed a unilocular, radiolucent lesion with well-defined margins and radiopaque halo in the mandibular body on the left side, involving second left premolar with a depth of inclusion of 14 mm. The lesion extended from the periapical region of left first premolar to the mesial root of left first molar, measuring 3.3 cm × 2.7 cm, suggesting the diagnosis of dentigerous cyst. It presented a 77.3° angle between the line that passes through the long axis of left second premolar and the plane bisecting the long axes of the adjacent teeth. There was a 17.5° angle formed between the line that follows the long axis of the mentioned tooth and the line that passes through the cementoenamel junction of the neighboring teeth (Figure 2).

**Figure 3:**
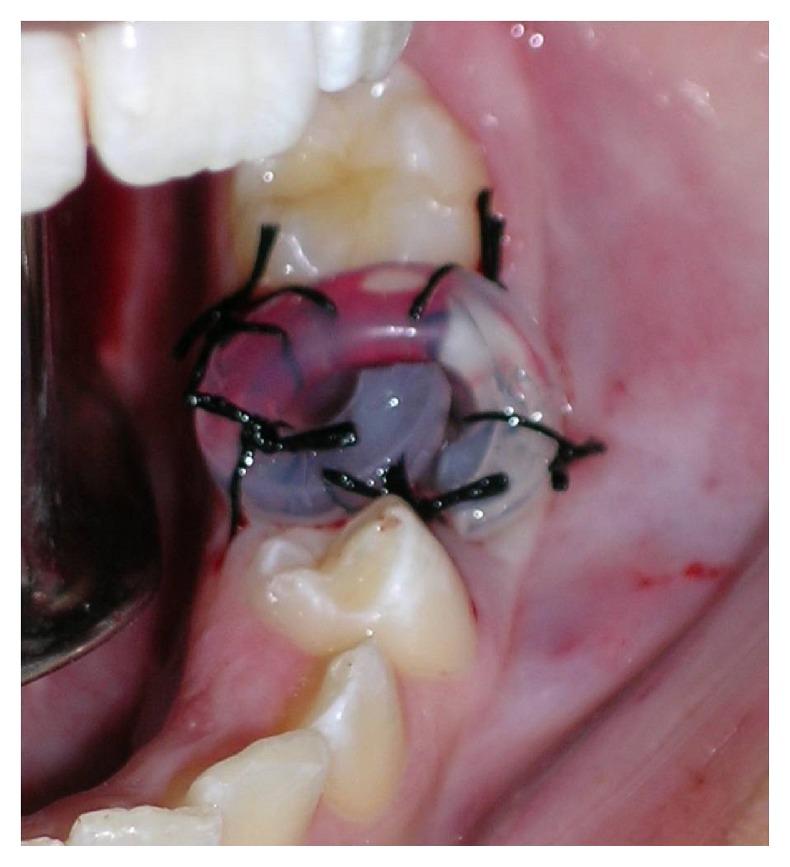
After extraction of second primary molar, the alveolus was communicated with the cyst, allowing the insertion of a decompression catheter.

**Figure 4:**
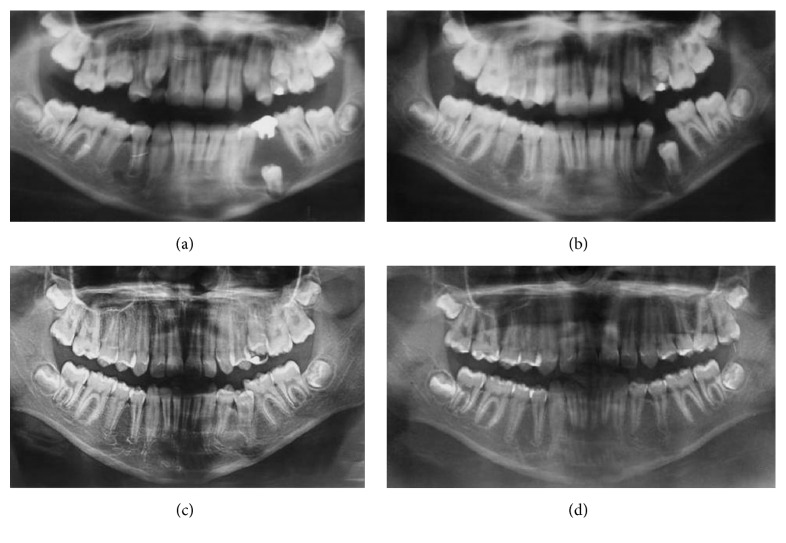
Initial aspect of the lesion (a). Note the extensive restoration of teeth 75 and 35 with incomplete rhizogenesis. Note the regression of the lesion and eruption of tooth 35 at 4 (b), 6 (c), and 11 months (d) after D/M.

**Figure 5:**
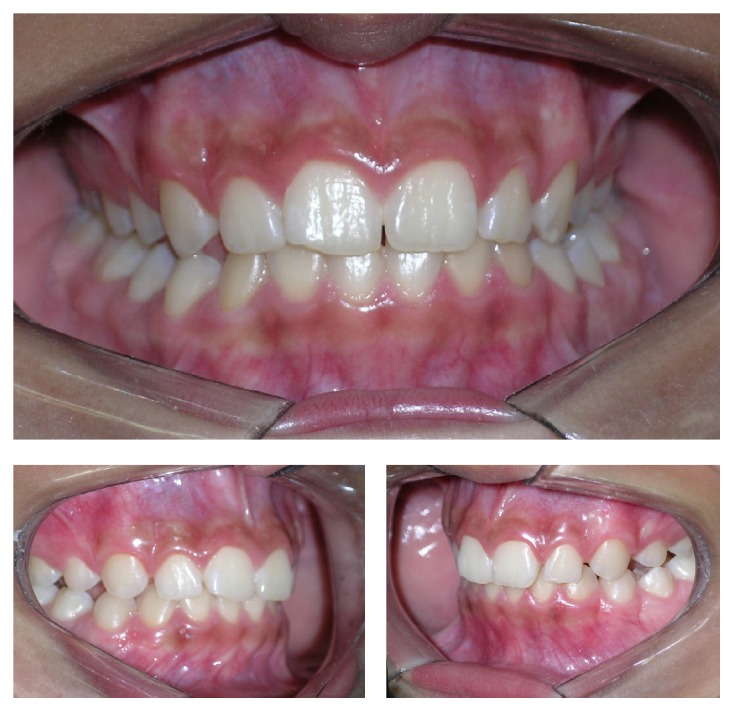
The patient was clinically followed up for a period of 1 year and 6 months, until complete regression of the lesion and eruption of tooth 35.

The patient was submitted to incisional biopsy under local anesthesia. The serum-blood colored surgical aspirate confirmed the hypothesis of cystic lesion. The opportunity was taken to remove tooth, the left second primary molar, and install a transalveolar silicone drain, which was removed after 3 weeks (Figure 3). The anatomopathological exam was conclusive for a dentigerous cyst associated with the presence of inflammatory infiltrate. The patient was clinically and radiographically followed up for a period of 1 year and 6 months, until complete regression of the lesion and eruption of tooth 35 (Figures 4 and 5).

## 3. Discussion

The DC is the most frequently occurring cyst among the odontogenic cysts, with the exception of the radicular cyst. There are few demographic studies about the subject involving populations in Brazil. In a retrospective study conducted in the Oral Pathology Service of FO-UFMG, in a period of 51 years, 19,064 oral biopsies were found. Of these, 2,812 cases (14.7%) were diagnosed as odontogenic cysts, with the DC showing a prevalence of 25.3% of these cases, being more common in young patients between the second and third decade of life [6]. Therefore, in spite of odontogenic cysts presenting a low incidence in children, the DC is of important clinical significance in pediatric dentistry, because it affects a significant number of child patients [7].

The DC is normally detected by chance, because it is asymptomatic. Therefore, they are generally detected due to a delay in the chronology of eruption of the tooth involved, causing deformation in the alveolar bone and expansion of the cortical bone, attaining important dimensions as a result of the delay in their diagnosis. In these cases, surgical treatment with minimal lesion to anatomic structures is fundamental. Postoperative defects of the facial structures in individuals exposed to treatment for enucleation of the cyst may bring about functional and esthetic problems, in addition to producing psychological consequences [8]. Therefore, enucleation is reserved for smaller lesions, or those in which it is not possible to save the tooth involved.

The M/D technique minimizes the risk of complications such as the loss of tooth germs, lesions to blood vessels and nerves, and loss of bone structures of the face. It is a simple, conservative surgical procedure that favors the eruption of displaced teeth and in the majority of cases results in good occlusion. By virtue of the foregoing, it is the treatment option for extensive lesions [9] and is well accepted in the treatment of cooperative pediatric patients, particularly when there is the possibility of preserving the tooth involved in the lesion and to allow eruption, thus diminishing the functional and esthetic damage to the patient. In the clinical case presented, enucleation would have resulted in the extraction of tooth 35 and probable lesion of the inferior alveolar/mental nerves, as well as fragilization of the left mandibular body, predisposing the patient to an eventual mandibular fracture. The patient developed without paresthesia or bone defects in the perilesional area.

An interesting topic concerning the case refers to the possibility of eruption of premolars involved with DC. Studies such as the ones of Hyomoto et al. [10], Fujii et al. [11], and Yahara et al. [12] have analyzed the factors that interfere in the spontaneous eruption of mandibular premolars associated with this cyst, based on the findings in panoramic radiographs (Table 1). Hyomoto et al. [10] furthermore observed that in the presence of inflammatory infiltrate the included teeth tend to erupt more easily, suggesting that the larger the quantity of inflammatory cells, the greater the predictability of eruption of the tooth associated with the cyst. This case ratifies the findings of the abovementioned authors, as the gradual eruption of the tooth involved in the lesion was observed in the radiographic images, until its functional occlusion occurred. The patient was 10 years of age, with 10 mm space present for eruption and the lesion involving tooth 35 with incomplete rhizogenesis and presence of inflammatory infiltrate. As regards angulation of the tooth involved, both by the method used by Hyomoto et al. [10] and Fujii et al. [11] and by the method of Yahara et al. [12], the case presented was shown to be favorable to eruption of the tooth involved, as may be observed in Figure 2. These findings may explain the fact of the extensive depth of inclusion not being sufficiently important, of itself alone, to result in the failure of spontaneous eruption of tooth 35.

Therefore, the characteristics of the inclusion are of fundamental importance in order to enable one to opt for an adequate treatment plan, with M/E being a simple technique to perform, with low postoperative morbidity, and easily accepted by the patient and his/her family members.

Among the treatment modalities proposed for DC, enucleation must be used in smaller lesions, in which there are no risks of injuring important anatomic structures and causing functional and esthetic damage, or when tooth eruption is improbable. The logic of the use of M/D follows the principle of inversion.

Spontaneous eruption of premolars associated with DC depends on a series of factors. Age, depth of inclusion, angulation, and degree of rhizogenesis are factors universally used. The quantity of space present between the neighboring teeth and the presence of inflammatory infiltrate may also be considered. Studies have confirmed that there is great predictability of the eruption of mandibular premolars related to dentigerous cysts [10–12]. Therefore, one must know the factors that have a positive influence on this process, so that one avoids unnecessarily sacrificing teeth and all the sequelae resulting from this fact. The time of intervention and decision making by the clinician is essential in order to establish a favorable prognosis, deciding on which treatment modality is appropriate after individual analysis of each case.

## Figures and Tables

**Table 1:** Factors related to spontaneous eruption of premolars associated with DC.

Factors analyzed	Hyomoto et al., 2003 [10]	Fujii et al., 2008 [11]	Yahara et al., 2009 [12]
Age	The smaller, the greater the chances of eruption	Under 10 years old	Mean age 9.8 years
Depth of inclusion	4.4 mm^*∗*^	Smaller than 5.1 mm^*∗*^	The smaller, the greater the chances of eruption
Angulation of tooth	20.4° ± 21.8°^*∗*^	Smaller than 25°^*∗*^	Close to 60.2°^*∗∗*^
Space present for eruption	Does not influence	Larger than 1 cm	Does not influence
Rhizogenesis	1/2	Incomplete	Does not influence

^*∗*^According to Hyomoto et al. [10] and Fujii et al. [11], it is considered the angle formed between the line that passes through the long axis of the included tooth and the plane bisecting the long axes of the neighboring teeth. The depth is considered as from the cementoenamel junction.

^*∗∗*^According to Yahara et al. [12], it is the measurement of the angle formed between the line that crosses the long axis of the tooth involved and the line that passes through the cementoenamel junction of the neighboring teeth.
